# Single-Step FRET-Based Detection of Femtomoles DNA

**DOI:** 10.3390/s19163495

**Published:** 2019-08-09

**Authors:** Kumar Sapkota, Anisa Kaur, Anoja Megalathan, Caleb Donkoh-Moore, Soma Dhakal

**Affiliations:** Department of Chemistry, Virginia Commonwealth University, Richmond, VA 23284, USA

**Keywords:** single molecule, FRET, DNA, sensing, single nucleotide polymorphism (SNP)

## Abstract

Sensitive detection of nucleic acids and identification of single nucleotide polymorphism (SNP) is crucial in diagnosis of genetic diseases. Many strategies have been developed for detection and analysis of DNA, including fluorescence, electrical, optical, and mechanical methods. Recent advances in fluorescence resonance energy transfer (FRET)-based sensing have provided a new avenue for sensitive and quantitative detection of various types of biomolecules in simple, rapid, and recyclable platforms. Here, we report single-step FRET-based DNA sensors designed to work via a toehold-mediated strand displacement (TMSD) process, leading to a distinct change in the FRET efficiency upon target binding. Using single-molecule FRET (smFRET), we show that these sensors can be regenerated in situ, and they allow detection of femtomoles DNA without the need for target amplification while still using a dramatically small sample size (fewer than three orders of magnitude compared to the typical sample size of bulk fluorescence). In addition, these single-molecule sensors exhibit a dynamic range of approximately two orders of magnitude. Using one of the sensors, we demonstrate that the single-base mismatch sequence can be discriminated from a fully matched DNA target, showing a high specificity of the method. These sensors with simple and recyclable design, sensitive detection of DNA, and the ability to discriminate single-base mismatch sequences may find applications in quantitative analysis of nucleic acid biomarkers.

## 1. Introduction

Over the years, a myriad of sensors have been developed for the specific detection of nucleic acids spanning a wide array of fields such as fundamental research, clinical diagnosis, and biotechnology [1,2,3]. With the advent of DNA nanotechnology, fluorescence resonance energy transfer (FRET)-based sensors have especially surged in popularity for their ability to detect a variety of targets including nucleic acids, proteins, and small molecules. Due to their simple and highly predictable design, ability to be regenerated through the toehold-mediated strand displacement (TMSD) process [4,5,6], and specificity towards pre-defined targets, DNA-based sensors have found applications in fluorescent hybridization assays [2,7,8], aptamer-based assays [7,9,10], electrochemical assays [3,11], surface enhanced Raman spectroscopy [12,13], and surface plasmon resonance analysis of biomolecules [14,15], just to name a few. Among these, bulk fluorescence methods are popular owing to their flexibility in sensor design, easy operation, and high sensitivity. However, they often suffer from high background, require large amounts of sample (typically nanomolar), and are difficult to multiplex [16,17].

In order to achieve better detection limits, bulk FRET strategies are often coupled with signal amplification steps such as exonuclease-III (ExoIII)-assisted amplification, rolling circle amplification (RCA), or hybridization chain reaction (HCR), all of which introduce a significant complexity to the assays [8,9,18]. For example, an enzymatic amplification requires a multi-step process and use of enzyme, which is costly and sensitive to reaction conditions such as temperature, pH, and enzyme inhibitors [8,18]. Other emerging examples demonstrating a sensitive detection of DNA down to low picomolar and high attomolar concentrations in an amplification-free format involve complex systems such as quantum dots, nanoparticles, cationic polymers, or microarrays [19,20,21,22,23,24,25,26,27,28,29]. In this regard, single-molecule FRET (smFRET) with the ability to measure many single molecules simultaneously offers several advantages over these complex systems and the bulk fluorescence assays [30]. For example, smFRET allows sensitive detection of DNA without the need for amplification and requires a dramatically small sample size compared to that of bulk fluorescence. Typically, the amount of DNA sensors required in smFRET is ~1000-fold less than in bulk approach (~200 µL of ~20 pM probes in single molecule vs. ~200 µL of ~30–60 nM probes in bulk). In addition, since smFRET experiments are performed in a flow cell with inlet and outlet channels, the surface-immobilized sensors can be readily regenerated in a few minutes by an in-situ TMSD process and buffer exchange [31].

Here, we report sensors that comprise two partially complementary DNA strands, each labeled with either a donor or an acceptor fluorophore. A probe sequence is partially hybridized to one of the strands and thus blocks the fluorophore-labeled strands from hybridizing to one another, giving a low FRET efficiency (low *E*_FRET_). In the presence of the target, the probe is unzipped via the TMSD process, allowing the labeled strands to hybridize and resulting in a high FRET efficiency (high *E*_FRET_). Using this strategy, we show that these sensors allow single-step, enzyme-free detection of femtomoles DNA without the need for target labeling and amplification. Such a low detection limit is difficult to achieve in bulk fluorescence methods without coupling them with a target amplification or a signal amplification step. In addition, our sensors demonstrate a detection limit either superior [32,33] or comparable [34,35,36] to molecular-beacon (MB) sensors.

In addition to sensitive and quantitative detection, another highly desirable feature of nucleic acid sensors is the ability to distinguish a point mutation called single nucleotide polymorphisms (SNPs), as more and more nucleic acid sequences have been found as biomarkers [37,38,39,40,41,42]. The single nucleotide substitutions of a genome are the most abundant genetic polymorphisms and are regarded as genetic markers for identifying inherited diseases and also for the development of drug candidates [8,43,44]. In this regard, a variety of methods spanning from single molecule to bulk analysis have been developed to distinguish SNPs [31,45,46], for example, enzyme assisted allele-specific biochemical reactions, electrochemical techniques, selective ligation with a fluorescent-modified DNA, synthetic nanopores containing molecular probes, etc. [47,48,49,50,51,52]. However, many of these methods require complicated sensor design, expensive enzymes, and/or labeling of the target in order to achieve a sensitive and specific detection. We show that, by tuning the length and the sequence of the toehold region of the probe strand, these sensors allow discrimination against non-target sequences with single point mutation. Although several examples have been reported in the literature for detection of DNA and discrimination of SNPs [52]. To the best of our knowledge, this study is the first-time demonstration of an smFRET-based approach that allows sensitive detection of DNA on a recyclable format with <10 femtomole detection limit and has the ability to discriminate SNPs. Overall, the single-molecule recyclable sensors developed here bear great potential for quantitative detection of nucleic acids and may find applications in biomarker and SNP analysis.

## 2. Materials and Methods

### 2.1. Materials

Magnesium chloride hexahydrate, tris(hydroxymethyl)-aminomethane (Tris), 6-hydroxy-2,5,7,8-tetramethylchroman-2-carboxylic acid (Trolox), protocatechuate 3,4-dioxygenase (PCD), acetic acid, and ethylenediaminetetraacetic acid disodium salt (EDTA) were purchased from Fisher Scientific. Biotinylated bovine serum albumin (bBSA) was purchased from Thermo Scientific. Protocatechuic acid (PCA) and streptavidin were purchased from VWR International, LLC. All DNA oligonucleotides were purchased from Integrated DNA Technologies (IDT), and primary stocks were prepared at 100 μM in filtered sterile water and stored at −20 °C.

### 2.2. Preparation of Sensors

Sensors were assembled from their constituent single stranded DNA (ssDNA) oligos (Table S1) at 1 µM concentrations via thermal annealing in 1× TAE-Mg Buffer (40 mM Tris, 20 mM acetic acid, 1 mM EDTA, 10 mM Mg^2+^, pH 7.4). The thermal annealing was carried out in a thermal cycler by heating the solution at 95 °C for 5 min and then slowly ramping the temperature down to 4 °C (Table S2), as described in our previous publications [31,53].

### 2.3. Bulk FRET Experiments

In order to determine if the sensors could be regenerated, bulk fluorescence measurements were conducted using a DeNovix FX-11 fluorimeter. A 525 nm light was used to excite Cy3, and the fluorescence emission intensities were collected at 565–650 nm and 665–740 nm ranges for green and red emissions, respectively. A 30 nM sensor construct in 1× TAE buffer and 10 mM MgCl_2_ was prepared, maintaining a total volume of 200 µL. Subsequently, 60 nM target DNA was added, and fluorescence emissions were recorded. The amount of target/probe was doubled in subsequent additions. The resulting fluorescence emissions, acceptor (Cy5) intensity (*I*_A_) and donor (Cy3) intensity (*I*_D_), were converted to *E*_FRET_ values using the following well-established equation: *E*_FRET_ = *I*_A_/(*I*_D_ + *I*_A_) [54,55]. All of the experiments were performed at room temperature (23 °C).

### 2.4. Surface-Functionalization of Flow Cell

The design and the construction of the flow cell are described elsewhere [53]. In single molecule experiments, before injection of the sample, the flow cell was functionalized via sequential incubation with 1 mg/mL biotinylated BSA (bBSA) for 5 min followed by incubation with 0.2 mg/mL streptavidin for 2 min. Afterwards, the flow cell was flushed with 300 µL of 1× TAE-Mg buffer to get rid of unbound molecules before collecting the FRET movies.

### 2.5. pTIRF Sample Preparation, Imaging, and Data Analysis

After mounting the functionalized flow cell on the stage of the prism-based total internal reflection fluorescence (pTIRF) microscope [56], a 200 µL solution of 20 pM biotinylated sensor construct prepared in an imaging buffer consisting of 10 mM MgCl_2_ in 1× TAE and an oxygen scavenging system (4 mM Trolox, 10 mM PCA, 100 nM PCD) was injected. Through Mg^2+^ titration experiments, we determined that these sensors performed optimally at 10 mM Mg^2+^ (Figure S1). Therefore, all of the single molecule FRET experiments were performed at 10 mM MgCl_2_. The flow cell was flushed with imaging buffer after 30 s of incubation in order to remove unbound sensor molecules. Data acquisition was performed at room temperature (23 °C) using Single.exe software, and post analysis was performed using IDL and MATLAB scripts, as described in our previous publications [31,53]. Using a 532 nm laser (power = ~32 mW), the Cy3 fluorophore was continuously excited throughout the movie (~200 s). Fluorescence emissions from Cy3 and Cy5 fluorophores were simultaneously recorded through green and red channels respectively (512 × 256 pixels) using an iXon Ultra EMCCD camera at a 100 ms time resolution. To confirm the presence of an active FRET pair in the sensor molecules, the red laser (λ = 639 nm, power = ~22 mW) was turned on after around 100 s of starting the movie. Those molecules showing evidence of both Cy3 and Cy5 with single-step photo-bleaching of the fluorophores were selected for further analysis. Finally, FRET histograms were plotted in Origin by binning the FRET data for the first 60 frames (all raw FRET data were combined from several molecules before binning) and fitted with one or a multi-peak Gaussian function. Although the movies were recorded for a long time window (~200 s), to include most of the molecules, we used the first 60 frames for binning the FRET data for histograms, as some molecules photobleach faster than others (Figure S2). The selected molecules were randomly assigned into three groups, and the three histograms obtained were used to estimate the mean FRET state, the FRET fractions (when relevant), and the standard deviations (SD). The number of replicates is identified in the figure legends, and the standard deviations are incorporated in the plots where applicable.

## 3. Results and Discussion

### 3.1. Sensor Design and Optimization

**Figure 1:**
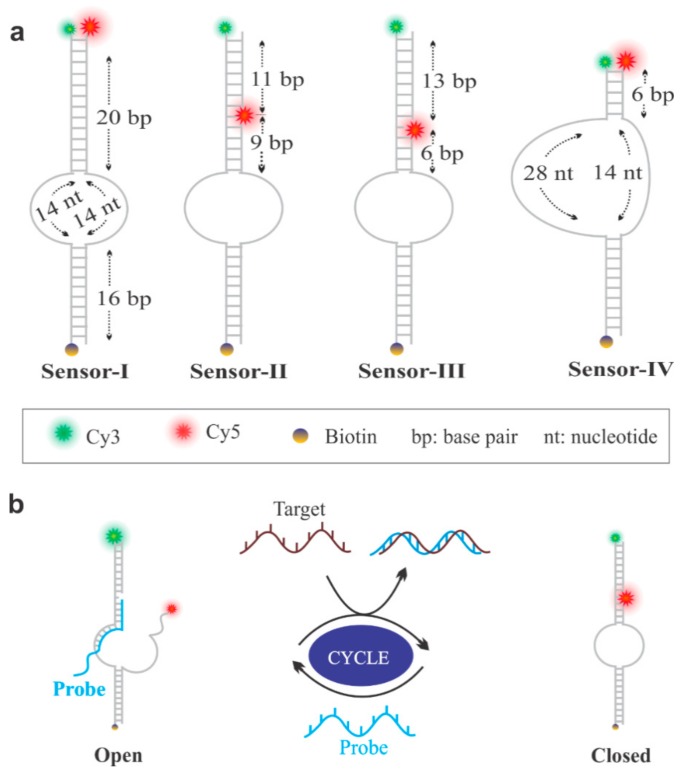
Sensor design and optimization. (**a**) Sensors with donor (Cy3) and acceptor (Cy5) fluorophores. The stem (defined as the number of base pairs formed between the Cy3- and the Cy5-labeled strands between the bulge (central loop) and the Cy5-labeled position) comprised 20, 9, 6, and 6 bp for sensors I, II, III, and IV, respectively. (**b**) Regeneration of sensors to an open (low *E*_FRET_) and a closed state (high *E*_FRET_) by addition of the probe and the target strand, respectively.

The sensor design and the experimental setup for this study are illustrated in Figure 1. All sensors were prepared by thermal annealing of three or four (depending on the design) single-stranded DNA (ssDNA) oligonucleotides (Table S1) in 1× TAE-Mg buffer (40 mM Tris, 20 mM acetic acid, 1 mM EDTA, 10 mM Mg^2+^, pH 7.4) using a temperature ramp from 95 to 4 °C, as described previously [31,53,56,57]. One of the constituent oligonucleotides was end-labeled with a biotin to enable surface-immobilization via biotin-streptavidin linkage (see Materials and Methods for detail.) 

**Figure 2:**
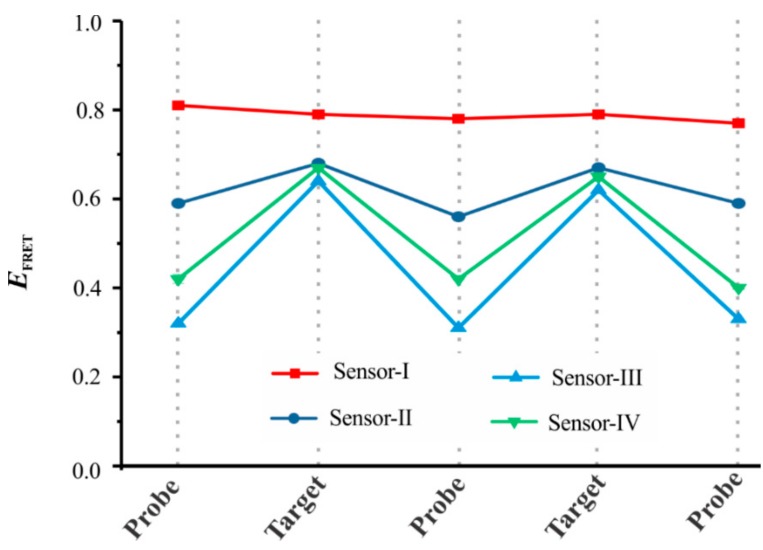
Analysis of the sensors’ ability to be regenerated. All four sensors (sensor-I to sensor-IV) at 30 nM concentration in 1 × TAE-Mg buffer were tested for bulk fluorescence resonance energy transfer (FRET) efficiency change upon addition of 60 nM target and 20 min incubation. For probe regeneration, two-fold excess of either the target or the probe strands were added in each subsequent steps. While sensor-I and sensor-II showed no to poor regeneration, sensors III and IV were fully regenerated (n = 3, errors bars are too small to be visible). All of the experiments were performed at room temperature (23 °C).

Additionally, a donor (Cy3) and an acceptor (Cy5) fluorophore were incorporated onto the oligonucleotides to enable detection by change in the FRET efficiency (*E*_FRET_). The sensors were assembled in their open conformation in the presence of a probe oligonucleotide that was partially complementary to the Cy3-labeled strand (Figure 1a), which allowed the sensor molecules to stay in their open conformation, resulting in a low FRET efficiency. However, in the presence of a target, the probe was displaced from the sensor via TMSD, allowing the hybridization of the fluorophore-labeled strands and resulting in a high *E*_FRET_ (Figure 1b). In this work, we started with sensor-I, and the rest of the sensors were evolved during the course of optimization to improve their ability to be regenerated (Figure 1b). For this, we tuned the length of the complementary region of the fluorophore-labeled strands just above the central bulge (Figure 1a). While the toehold length of the probe was designed to be 15-nt for all of the sensors, the complementary region that bound to the fluorophore-labeled strand varied from design to design, as illustrated in Figure 1a. The bulk *E*_FRET_ analysis of sensor-I showed that the sensor assumed a closed conformation which was not able to open (Figure 2), suggesting that the 20 bp stem was too long to be invaded by the probe. The reduction of the stem length to 9 bp (sensor-II) improved sensor regeneration; however, the *E*_FRET_ change before and after the addition of the target was not significant. Further shortening of the stem length to 6 bp made the sensors fully recyclable (sensor-III and IV) for multiple rounds (Figure 2). All of these cycling data were collected at the optimal concentration of Mg^2+^ (10 mM), which we determined by bulk *E*_FRET_ analysis of sensors at various concentrations of Mg^2+^ (Figure S1).

### 3.2. Single Molecule Analysis of DNA Sensors

**Figure 3:**
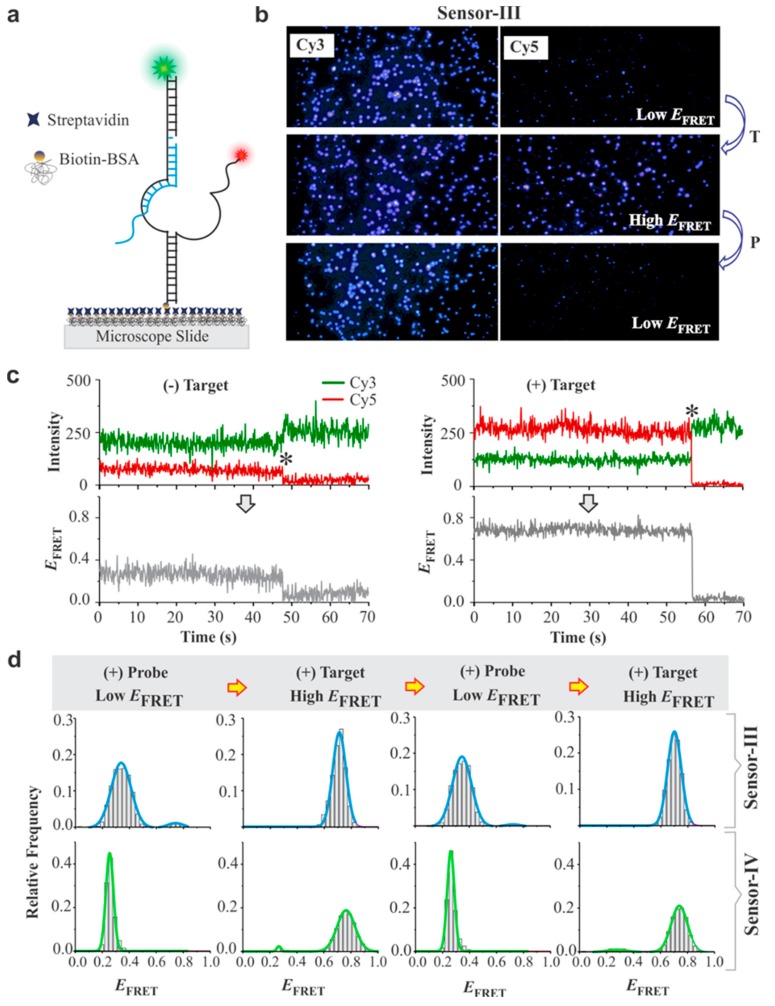
Single molecule analysis of DNA sensors. (**a**) Experimental setup for the prism-based total internal reflection fluorescence (pTIRF) microscopy analysis of sensors. The quartz slide was coated with biotin bovine serum albumin (bBSA) and then streptavidin. The sensor molecules were immobilized on the slide via biotin–streptavidin interaction. (**b**) Fluorescence images of surface-immobilized sensor-III before (top) and after addition of target DNA (middle). The image of the same microscope slide after addition of probe (bottom). (**c**) Typical single molecule traces of sensor-III. Left: open conformation (low-*E*_FRET_ state); right: closed conformation (high-*E*_FRET_ state). Top panels display fluorescence intensities of the donor (Cy3: green) and the accepter (Cy5: red) fluorophore and the bottom panel depicts FRET efficiencies calculated from the corresponding intensity–time traces. The asterisks indicate the photobleaching events of Cy5 fluorophores. (**d**) single-molecule FRET (smFRET) histograms of sensors III and IV each switching between the low- and the high-*E*_FRET_ conformations after alternate addition of an excess (1 μM) probe (P) and target (T). Each *E*_FRET_ histogram was prepared from 95–110 molecules.

To make our sensors suitable for single molecule analysis using a prism-based TIRF (pTIRF) microscope, a biotin was incorporated to the 5’-end of the Cy3-labeled strand (Figure 3a). The flow cells were prepared and modified with biotin-BSA and streptavidin before immobilization of the sensors, as described in the Materials and Methods [31,53,56]. Briefly, to immobilize sensors on the microscope slide, 20 pM sensor was added, and unbound species were washed with imaging buffer containing oxygen scavenging system (OSS) to limit fluorophore blinking and photobleaching upon laser illumination [58,59]. The microscope slide was illuminated with a 532 nm laser to create an evanescent field to excite the Cy3 fluorophores, as described previously (Figure 3b) [53,56]. The fluorescence intensity–time traces of both the Cy3 and the Cy5 fluorophores were recorded at 10 frames per second (≈ 100 ms camera integration time) while the field of view was illuminated by the green laser (Figure 3c). The presence of the fully assembled sensor molecules was confirmed either by anti-correlation of Cy3/Cy5 intensities (indicated by asterisk in Figure 3c) or by increase in the Cy5 intensity when the red laser (639 nm) was turned on to allow direct excitation of the Cy5 fluorophore towards the end of data acquisition. Only the sensor molecules that showed clear evidence of both Cy3 and Cy5 fluorophores were selected for subsequent FRET analysis. IDL and MATLAB programs were employed to process the FRET movies (see Materials and Methods), and the FRET efficiencies were calculated using the equation, *I*_A_/(*I*_D_ + *I*_A_), where *I*_A_ and *I*_D_ represent the background-corrected fluorescence intensities of the donor and the acceptor fluorophores, respectively [54,55]. 

The smFRET analyses showed an intensity switch between Cy3 and Cy5 emissions when the target was added (Figure 3c, higher Cy5 intensity in the presence of the target and hence higher FRET efficiency). This assignment was clear when we binned the smFRET data to construct histograms after combining the raw FRET data of several single molecules (Figure 3d). The ability of sensors III and IV to regenerate was confirmed by switching between low- and high-*E*_FRET_ upon alternate addition of probe (P) and target (T) for multiple rounds (Figure 3d). This ability of the sensor was also demonstrated by using a probe that bound to the Cy5-labeled strand instead of Cy3-labeled strand (Figure S3).

### 3.3. Analytical Sensitivity of DNA Sensors

**Figure 4:**
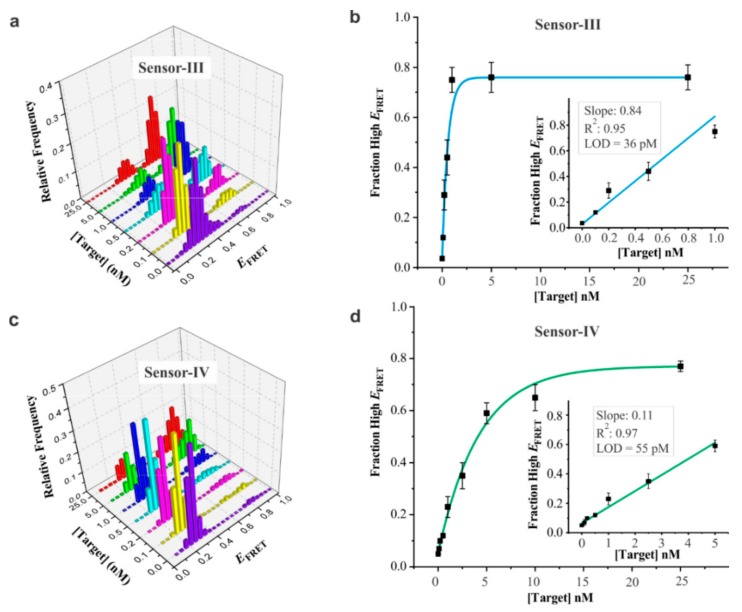
Analytical sensitivity of DNA sensors. (**a**) smFRET histograms of sensor-III for different concentrations of the target (0, 0.1, 0.2, 0.5, 1, 5, and 25 nM). The 0 nM data represent control experiment in the absence of the target. (**b**) Calibration curve of sensor-III depicting fraction of high-*E*_FRET_ population against target concentration. High-*E*_FRET_ population was determined from the two-peak Gaussian fitting of the histograms in Figure 3a. Inset displays the linear region of the full titration curve. R^2^ value obtained from linear fitting was 0.95, and the limit of detection (LOD) was 36 pM, as determined using the equation, LOD = (3 × SD_blank_)/slope, where SD_blank_ represents the standard deviation of high-E_FRET_ fraction in the absence of the target. (**c**) smFRET histograms of sensor-IV for different concentrations of the target. (**d**) Calibration curve of sensor-IV. Inset depicts the linear fitting region. R^2^ value from linear fit was 0.97, and the detection limit was calculated to be 55 pM. Error bars in (**b**) and (**d**) represent the standard deviations (SD), n = 3. Each *E*_FRET_ histogram was prepared from 90–110 molecules.

To determine the analytical sensitivity of the sensors, we acquired smFRET histograms (Figure 4) for both sensor-III (Figure 4a) and sensor-IV (Figure 4c) in the absence (control) and in the presence of different concentrations of the target. When we compared the area under the curve (AUC) for the high-*E*_FRET_ population to that of the low-*E*_FRET_ population, we observed a correlation between the high-*E*_FRET_ fraction and the concentration of the target. The plots showed a linear increase of the high-*E*_FRET_ fraction up to approximately 1.0 nM and 10 nM target for sensor-III and sensor-IV, respectively, after which the curves were plateaued at higher concentrations (Figure 4b,d). The calculated limit of detections (LOD, insets), defined as 3 × SD_blank_/slope, were 36 and 55 pM, respectively, for sensors III and IV. Given the flow cell volume of ~150 µL, the detection limits of 36 and 55 pM translated to 5.4 and 8.3 femtomoles, respectively. These results show that both of these sensors are suitable for a sensitive detection of DNA in an amplification-free format without any added complexity. These detection limits are either superior or comparable to the molecular beacon approach for nucleic acids detection without pre-amplification of targets [32,33,34,35,36].

Interestingly, compared to sensor-III, we observed a much shallower response of sensor-IV to the target concentration (compare the binding curves in Figure 4b,d). We attributed this observation to difference in sensor designs. The two sensors are different in terms of the ssDNA length in the bulge region (Figure 1a). Sensor-III has a 13bp duplex portion on the Cy3-labeled strand, leading to a relatively higher E_FRET_ in its open state. However, compared to sensor-III, sensor-IV assumed a slightly higher E_FRET_ in its closed state, which was due to the close proximity of the fluorophores (Figure 1a) when the two strands hybridized. Further, from the calibration curves, we noticed that sensor-III was more sensitive to change in the concentration of the target, whereas sensor-IV exhibited a higher dynamic range (36 pM–1 nM for sensor-III vs. 55 pM–10 nM for sensor-IV). While these sensors exhibited dynamic ranges of one to two orders of magnitude, the significant difference (10-fold) in the dynamic range is particularly interesting for the rational design of sensors with desired dynamic ranges and the implementation of them according to specific needs.

### 3.4. Specificity of DNA Sensors

**Figure 5:**
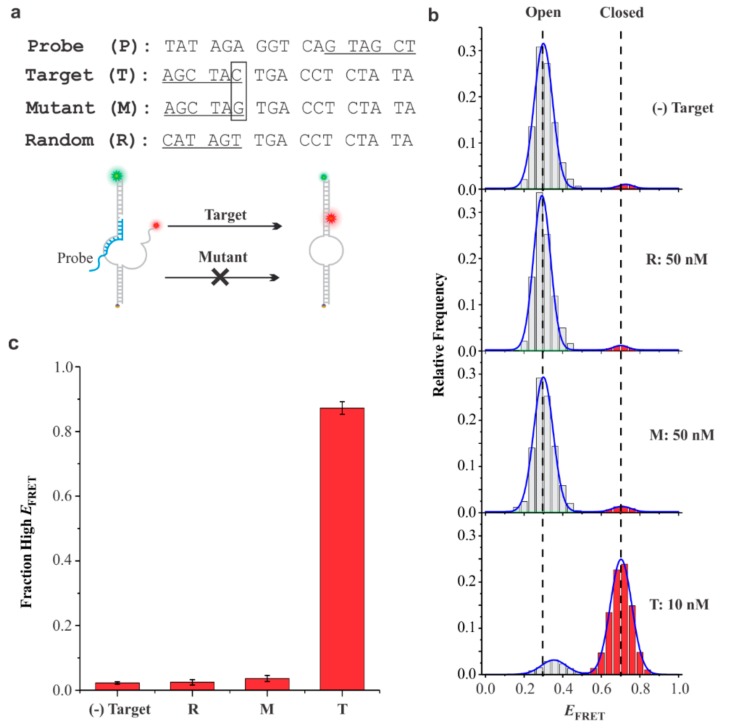
Discrimination of single nucleotide mismatch from fully matched target. (**a**) The sequences of probe (P), target (T), mutant (M), and random (R) used for specificity analysis using sensor-III. Target refers to perfect complementary sequence. Mutant refers to a single nucleotide mutated target, whereas random refers to a target with fully randomized toehold region. The toehold regions are underlined, and the point mutation is identified with a box. (**b**) The smFRET histogram without the target (top panel) followed by the smFRET histograms after incubation with random, mutant, and fully matched target sequences. Almost none high-*E*_FRET_ population for random and single-base mismatch target demonstrated that these sequences were not able to displace the probe. The *E*_FRET_ histograms were prepared from ~200 molecules. (**c**) Comparison of high-*E*_FRET_ fraction under different conditions. Histogram depicts that high-*E*_FRET_ fractions for both the mutant and the target were similar to the background signal (2.3 ± 0.4%). However, in the presence of the target, the high-*E*_FRET_ fraction increased significantly (87%). Error bars represent the standard deviations (SD), n = 3.

To evaluate the specificity of our sensors, we used sensor-III and examined whether the sensor was specific enough to discriminate targets that differed by a single nucleotide mismatch (Figure 5). 

In this regard, we took advantage of the literature that optimized the toehold length and mismatch locations in TMSD processes to achieve high specificity of a fully matched target against single nucleotide mismatches [60,61]. These studies demonstrated that six-base toeholds with inner-end mutations were highly effective in discriminating single nucleotide mismatches. When we tested our sensors with a probe containing six-base toehold (Figure 5a), the fully matched target was easily detected, whereas the same analysis with a single-nucleotide mismatch at the inner-end position was not detected (Figure 5b,c), even at a five-fold excess, demonstrating a high specificity towards the complementary targets. These results demonstrated that the sensors developed here bear promising real life applications.

## 4. Conclusions

It has been well established that the detection and the quantification of specific nucleic acid sequences are important in clinical diagnostics, as many nucleic acid sequences have been found to be biomarkers for specific diseases. Despite a myriad of methods available for nucleic acid analysis, a vast majority of them require complicated designing of the platforms, expensive enzymes, or labeling of the target DNA in order to achieve a sensitive and specific detection. We developed FRET-based sensors, which allow sensitive detection of a fully matched target down to femtomoles DNA. These sensors with a multitude of features, such as straightforward design, sensitive detection of DNA, large dynamic range, and ability to cycle and discriminate single-base mismatch sequences, may find applications in quantitative analysis of nucleic acid biomarkers.

## Supplementary Materials

The following are available online at https://www.mdpi.com/1424-8220/19/16/3495/s1, Table S1: DNA sequences, Table S2: Thermal annealing program, Figure S1: Effect of Mg^2+^ concentration on sensor performance, Figure S2: Typical single-molecule traces, Figure S3: Single-molecule analysis of sensor-III with an alternative way of target binding.

## References

[1] Liu J., Cao Z., Lu Y. (2009). Functional Nucleic Acid Sensors. Chem. Rev..

[2] Nimse S.B., Sonawane M.D., Song K.-S., Kim T. (2016). Biomarker Detection Technologies and Future Directions. Analyst.

[3] Chikkaveeraiah B.V., Bhirde A.A., Morgan N.Y., Eden H.S., Chen X. (2012). Electrochemical Immunosensors for Detection of Cancer Protein Biomarkers. ACS Nano.

[4] Srinivas N., Ouldridge T.E., Šulc P., Schaeffer J.M., Yurke B., Louis A.A., Doye J.P.K., Winfree E. (2013). On the Biophysics and Kinetics of Toehold-Mediated DNA Strand Displacement. Nucleic Acids Res..

[5] Srinivas N., Parkin J., Seelig G., Winfree E., Soloveichik D. (2017). Enzyme-Free Nucleic Acid Dynamical Systems. Science.

[6] Li J., Johnson-Buck A., Yang Y.R., Shih W.M., Yan H., Walter N.G. (2018). Exploring the Speed Limit of Toehold Exchange with a Cartwheeling DNA Acrobat. Nat. Nanotechnol..

[7] Yang K., Yang Y., Zhang C. (2013). Single-Molecule FRET for Ultrasensitive Detection of Biomolecules. NanoBioImaging.

[8] Ling Y., Zhang X.F., Chen X.H., Liu L., Wang X.H., Wang D.S., Li N.B., Luo H.Q. (2018). A Dual-Cycling Biosensor for Target DNA Detection Based on the Toehold-Mediated Strand Displacement Reaction and Exonuclease III Assisted Amplification. New J. Chem..

[9] Deng B., Lin Y., Wang C., Li F., Wang Z., Zhang H., Li X.-F., Le X.C. (2014). Aptamer Binding Assays for Proteins: The Thrombin Example—A Review. Anal. Chim. Acta.

[10] Song Q., Wang R., Sun F., Chen H., Wang Z., Na N., Ouyang J. (2017). A Nuclease-Assisted Label-Free Aptasensor for Fluorescence Turn-on Detection of ATP Based on the in Situ Formation of Copper Nanoparticles. Biosens. Bioelectron..

[11] Labib M., Khan N., Ghobadloo S.M., Cheng J., Pezacki J.P., Berezovski M.V. (2013). Three-Mode Electrochemical Sensing of Ultralow MicroRNA Levels. J. Am. Chem. Soc..

[12] Barhoumi A., Zhang D., Tam F., Halas N.J. (2008). Surface-Enhanced Raman Spectroscopy of DNA. J. Am. Chem. Soc..

[13] Panikkanvalappil S.R., Mackey M.A., El-Sayed M.A. (2013). Probing the Unique Dehydration-Induced Structural Modifications in Cancer Cell DNA Using Surface Enhanced Raman Spectroscopy. J. Am. Chem. Soc..

[14] Law W.-C., Yong K.-T., Baev A., Prasad P.N. (2011). Sensitivity Improved Surface Plasmon Resonance Biosensor for Cancer Biomarker Detection Based on Plasmonic Enhancement. ACS Nano.

[15] Krishnan S., Mani V., Wasalathanthri D., Kumar C.V., Rusling J.F. (2011). Attomolar Detection of a Cancer Biomarker Protein in Serum by Surface Plasmon Resonance Using Superparamagnetic Particle Labels. Angew. Chem. Int. Ed..

[16] Gui Z., Wang Q.B., Li J.C., Zhu M.C., Yu L.L., Xun T., Yan F., Ju H.X. (2016). Direct Detection of Circulating Free DNA Extracted from Serum Samples of Breast Cancer Using Locked Nucleic Acid Molecular Beacon. Talanta.

[17] Huang S., Qiu H., Xiao Q., Huang C., Su W., Hu B. (2013). A Simple QD–FRET Bioprobe for Sensitive and Specific Detection of Hepatitis B Virus DNA. J. Fluoresc..

[18] Bi S., Yue S., Zhang S. (2017). Hybridization chain reaction: A versatile molecular tool for biosensing, bioimaging, and biomedicine. Chem. Soc. Rev..

[19] Cannon B., Campos A.R., Lewitz Z., Willets K.A., Russell R. (2012). Zeptomole Detection of DNA Nanoparticles by Single-Molecule Fluorescence with Magnetic Field-Directed Localization. Anal. Biochem..

[20] Mayr R., Haider M., Thünauer R., Haselgrübler T., Schütz G.J., Sonnleitner A., Hesse J. (2016). A Microfluidic Platform for Transcription-and Amplification-Free Detection of Zepto-Mole Amounts of Nucleic Acid Molecules. Biosens. Bioelectron..

[21] Li L., Li X., Li L., Wang J., Jin W. (2011). Ultra-Sensitive DNA Assay Based on Single-Molecule Detection Coupled with Fluorescent Quantum Dot-Labeling and Its Application to Determination of Messenger RNA. Anal. Chim. Acta.

[22] Hesse J., Jacak J., Kasper M., Regl G., Eichberger T., Winklmayr M., Aberger F., Sonnleitner M., Schlapak R., Howorka S. (2006). RNA Expression Profiling at the Single Molecule Level. Genome Res..

[23] Anazawa T., Matsunaga H., Yeung E.S. (2002). Electrophoretic Quantitation of Nucleic Acids without Amplification by Single-Molecule Imaging. Anal. Chem..

[24] Doré K., Dubus S., Ho H.-A., Lévesque I., Brunette M., Corbeil G., Boissinot M., Boivin G., Bergeron M.G., Boudreau D. (2004). Fluorescent Polymeric Transducer for the Rapid, Simple, and Specific Detection of Nucleic Acids at the Zeptomole Level. J. Am. Chem. Soc..

[25] Zhang Y., Ning X., Mao G., Ji X., He Z. (2018). Fluorescence turn-on detection of target sequence DNA based on silicon nanodot-mediated quenching. Anal. Bioanal. Chem..

[26] Shamsipur M., Nasirian V., Mansouri K., Barati A., Veisi A., Kashanian S. (2017). A highly sensitive quantum dots-DNA nanobiosensor based on fluorescence resonance energy transfer for rapid detection of nanomolar amounts of human papillomavirus 18. J. Pharm. Biomed. Anal..

[27] Joda H., Moutsiopoulou A., Stone G., Daunert S., Deo S. (2018). Design of Gaussia luciferase-based bioluminescent stem-loop probe for sensitive detection of HIV-1 nucleic acids. Analyst.

[28] Chen M., Hou C., Huo D., Bao J., Fa H., Shen C. (2016). An electrochemical DNA biosensor based on nitrogen-doped graphene/Au nanoparticles for human multidrug resistance gene detection. Biosens. Bioelecton..

[29] Esfandiari L., Lorenzini M., Kocharyan G., Monbouquette H.G., Schmidt J.J. (2014). Sequence-Specific DNA Detection at 10 FM by Electromechanical Signal Transduction. Anal. Chem..

[30] Gooding J.J., Gaus K. (2016). Single-Molecule Sensors: Challenges and Opportunities for Quantitative Analysis. Angew. Chem. Int. Ed..

[31] Kaur A., Sapkota K., Dhakal S. (2019). Multiplexed Nucleic Acid Sensing with Single-Molecule FRET. ACS Sens..

[32] Su Q., Wesner D., Schönherr H., Nöll G. (2014). Molecular Beacon Modified Sensor Chips for Oligonucleotide Detection with Optical Readout. Langmuir.

[33] Mahani M., Mousapour Z., Divsar F., Nomani A., Ju H. (2019). A Carbon Dot and Molecular Beacon Based Fluorometric Sensor for the Cancer Marker MicroRNA-21. Microchim. Acta.

[34] Liu Y., Kannegulla A., Wu B., Cheng L.-J. (2018). Quantum Dot Fullerene-Based Molecular Beacon Nanosensors for Rapid, Highly Sensitive Nucleic Acid Detection. ACS Appl. Mater. Interfaces.

[35] Ho S.-L., Chan H.-M., Ha A.W.-Y., Wong R.N.-S., Li H.-W. (2014). Direct Quantification of Circulating MiRNAs in Different Stages of Nasopharyngeal Cancerous Serum Samples in Single Molecule Level with Total Internal Reflection Fluorescence Microscopy. Anal. Chem..

[36] Zhang C.-Y., Chao S.-Y., Wang T.-H. (2005). Comparative Quantification of Nucleic Acids Using Single-Molecule Detection and Molecular Beacons. Analyst.

[37] Schwarzenbach H., Hoon D.S.B., Pantel K. (2011). Cell-Free Nucleic Acids as Biomarkers in Cancer Patients. Nat. Rev. Cancer.

[38] Mo M.-H., Chen L., Fu Y., Wang W., Fu S.W. (2012). Cell-Free Circulating MiRNA Biomarkers in Cancer. J. Cancer.

[39] Bartels C.L., Tsongalis G.J. (2009). MicroRNAs: Novel Biomarkers for Human Cancer. Clin. Chem..

[40] Shin V.Y., Siu J.M., Cheuk I., Ng E.K.O., Kwong A. (2015). Circulating Cell-Free MiRNAs as Biomarker for Triple-Negative Breast Cancer. Br. J. Cancer.

[41] Gilad S., Meiri E., Yogev Y., Benjamin S., Lebanony D., Yerushalmi N., Benjamin H., Kushnir M., Cholakh H., Melamed N. (2008). Serum MicroRNAs Are Promising Novel Biomarkers. PLoS ONE.

[42] Miranda K.C., Bond D.T., McKee M., Skog J., Păunescu T.G., Da Silva N., Brown D., Russo L.M. (2010). Nucleic Acids within Urinary Exosomes/Microvesicles Are Potential Biomarkers for Renal Disease. Kidney Int..

[43] Kolpashchikov D.M. (2008). Split DNA Enzyme for Visual Single Nucleotide Polymorphism Typing. J. Am. Chem. Soc..

[44] Musumeci D., Platella C., Riccardi C., Moccia F., Montesarchio D. (2017). Fluorescence Sensing Using DNA Aptamers in Cancer Research and Clinical Diagnostics. Cancers.

[45] Zhou X., Yao D., He M., Xiao S., Liang H. (2018). Optimizing the Toehold Strategy of On-Chip Nucleic Acid Hybridization Probe for the Discrimination of Single Nucleotide Polymorphism. Langmuir.

[46] Zhang D.Y., Chen S.X., Yin P. (2012). Optimizing the Specificity of Nucleic Acid Hybridization. Nat. Chem..

[47] Kim W.J., Sato Y., Akaike T., Maruyama A. (2003). Cationic Comb-Type Copolymers for DNA Analysis. Nat. Mater..

[48] Huh Y.S., Lowe A.J., Strickland A.D., Batt C.A., Erickson D. (2009). Surface-Enhanced Raman Scattering Based Ligase Detection Reaction. J. Am. Chem. Soc..

[49] Wang X., Lou X., Wang Y., Guo Q., Fang Z., Zhong X., Mao H., Jin Q., Wu L., Zhao H. (2010). QDs-DNA Nanosensor for the Detection of Hepatitis B Virus DNA and the Single-Base Mutants. Biosens. Bioelectron..

[50] Howorka S., Cheley S., Bayley H. (2001). Sequence-Specific Detection of Individual DNA Strands Using Engineered Nanopores. Nat. Biotechnol..

[51] Kohli P., Harrell C.C., Cao Z., Gasparac R., Tan W., Martin C.R. (2004). DNA-Functionalized Nanotube Membranes with Single-Base Mismatch Selectivity. Science.

[52] Sobrino B., Brión M., Carracedo A. (2005). SNPs in Forensic Genetics: A Review on SNP Typing Methodologies. Forensic Sci. Int..

[53] Gibbs D.R., Dhakal S. (2018). Single-Molecule Imaging Reveals Conformational Manipulation of Holliday Junction DNA by the Junction Processing Protein RuvA. Biochemistry.

[54] Roy R., Hohng S., Ha T. (2008). A Practical Guide to Single-Molecule FRET. Nat. Methods.

[55] Ha T. (2001). Single-Molecule Fluorescence Resonance Energy Transfer. Methods.

[56] Gibbs D.R., Kaur A., Megalathan A., Sapkota K., Dhakal S. (2018). Build Your Own Microscope: Step-By-Step Guide for Building a Prism-Based TIRF Microscope. Methods Protoc..

[57] Megalathan A., Cox B.D., Wilkerson P.D., Kaur A., Sapkota K., Reiner J.E., Dhakal S. (2019). Single-Molecule Analysis of i-Motif within Self-Assembled DNA Duplexes and Nanocircles. Nucleic Acids Res..

[58] Aitken C.E., Marshall R.A., Puglisi J.D. (2008). An Oxygen Scavenging System for Improvement of Dye Stability in Single-Molecule Fluorescence Experiments. Biophys. J..

[59] Fu J., Yang Y.R., Dhakal S., Zhao Z., Liu M., Zhang T., Walter N.G., Yan H. (2016). Assembly of Multienzyme Complexes on DNA Nanostructures. Nat. Protoc..

[60] Wang X., Zou M., Huang H., Ren Y., Li L., Yang X., Li N. (2013). Gold Nanoparticle Enhanced Fluorescence Anisotropy for the Assay of Single Nucleotide Polymorphisms (SNPs) Based on Toehold-Mediated Strand-Displacement Reaction. Biosens. Bioelectron..

[61] Long J.-B., Liu Y.-X., Cao Q.-F., Guo Q.-P., Yan S.-Y., Meng X.-X. (2015). Sensitive and Enzyme-Free Detection for Single Nucleotide Polymorphism Using Microbead-Assisted Toehold-Mediated Strand Displacement Reaction. Chin. Chem. Lett..

