# Supplementary material for: Single-Step FRET-Based Detection of Femtomoles DNA
Source: Sensors (Basel). 2019 Aug 9;19(16):3495. doi: 10.3390/s19163495 (PMC6719117; doi:10.3390/s19163495)
Supplement: Supplementary file 1 [file sensors-19-03495-s001.pdf]

sensors

Single-step FRET-based detection of femtomoles DNA

Kumar Sapkota, Anisa Kaur, Anoja Megalathan, Caleb Donkoh-Moore and Soma Dhakal *

Department of Chemistry, Virginia Commonwealth University, Richmond, VA 23284, USA
* Correspondence: sndhakal@vcu.edu

Table S1. Sequences for all the oligonucleotides used in assembling DNA constructs. All biotin- and fluorophore-modified DNA oligos were HPLC purified. S-I, S-II, S-III, and S-IV represent sensor-I through IV. The bulge region (internal loop) sequences are bolded. 'TEG' represents tetraethyleneglycol spacer between biotin and DNA, which helps avoid hindrance. The example schematics on the next page illustrate the naming of various strands used in making these sensors.

**Strand Name**	**Sequence (5'-3')**
Cy3 strand	Biotin-TEG/TGG AAC TCA CTA CTC G**AT TAG TGT ATG ACC** TCT ATA TGA GAG CTT CTG AT/Cy3
S-I Cy5 strand	Cy5/ TTC AGA AGC TCT CAT ATA GA**A TTA TAT TAT ATT A**CT AGT AGT GAG TTC CA
S-II Cy5 strand	Cy5/TCA TAT AGA **ATT ATA TTA TAT TA**C GAG TAG TGA GTT CCA
S-III Cy5 strand	Cy5/TAT AGA **ATT ATA TTA TAT TA**C GAG TAG TGA GTT CCA
S-IV Cy5 strand	Cy5/AT CAG A**TT TAT ATT ATA TTA** CGA GTA GTG AGT TCC A
S-II top strand	ATC AGA AGC TC
S-III top strand	ATC AGA AGC TCT C
S-I Probe	ATC TGA ACC TCT CAT ATA GAG GTC ATA CAC ATA GCA CTT TTT ACA
S-I Target	TGT AAA AAG TGC TAT GTG TAT GAC CTC TAT ATG AGA G
S-II & S-III Probe	TCA TAT AGA GGT CAA TAG CAC TTT TTA CA
S-II & S-III Target	TGT AAA AAG TGC TAT TGA CCT CTA TAT GA
S-IV Probe	ACA TTT TTC ACG ATA AAT ATA AAT CTG AG
S-IV Target	ATC AGA TTT ATA TTT ATC GTG AAA AAT GT
**Sequences used for SNP experiments:** (C to G mismatch is shown in red and bolded)	
Probe	TAT AGA GGT CAG TAG CT
Target	AGC TA**C** TGA CCT CTA TA
Mutant	AGC TA**G** TGA CCT CTA TA
Random	CAT AGT TGA CCT CTA TA

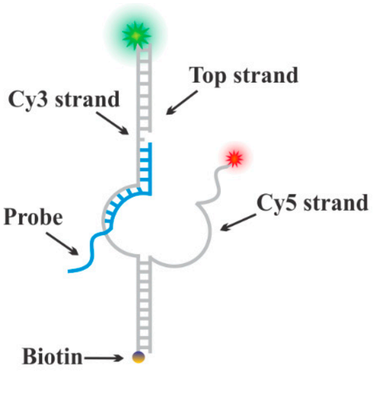

Sensor-III

Figure S1. Example schematics of sensors with strand name matching the Table S1.

Table 2. Thermal annealing program for the assembly of DNA constructs. The thermal annealing was carried out by ramping the temperature of the solution from 95 °C to 4 °C in a thermal cycler as described. [1,2.]

Temperature (°C)	Time (min)
95	5
93	5
90	5
88	5
86	5
84	5
82	5
80	5
78	5
76	5
72	5
68	5
64	5
60	5
56	5
52	5
48	5
44	5
40	5
36	5
32	5
28	5
24	5
4	hold

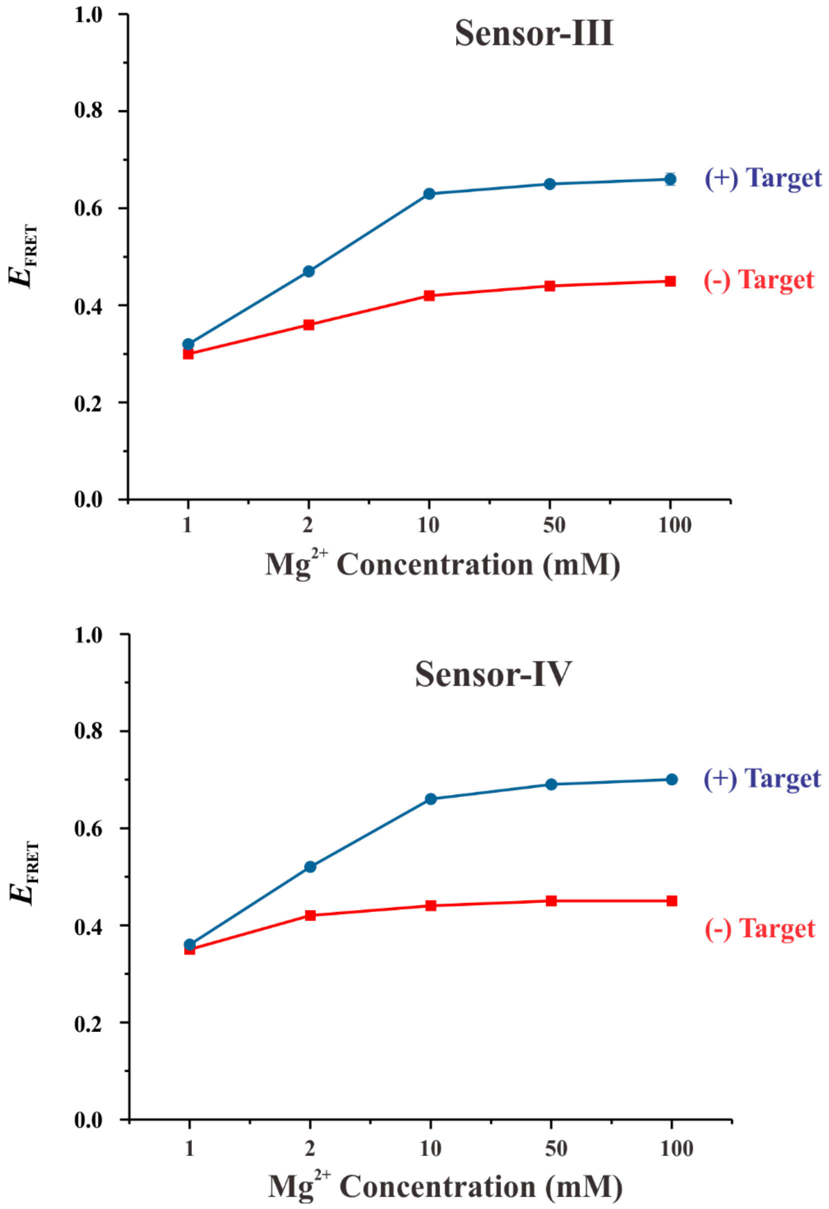

Figure S2. Mg^{2+}-dependent switching of sensors determined by bulk FRET analysis for sensor-III (top) and sensor-IV (bottom). In the presence of target, the FRET efficiency was gradually increased until 10 mM Mg^{2+} and was plateaued at higher concentration for both of the sensors. The slight increase in the FRET efficiency even in the absence of target suggested a compaction of single-stranded oligos due to electrostatic shielding of negative charge on DNA.[2.]

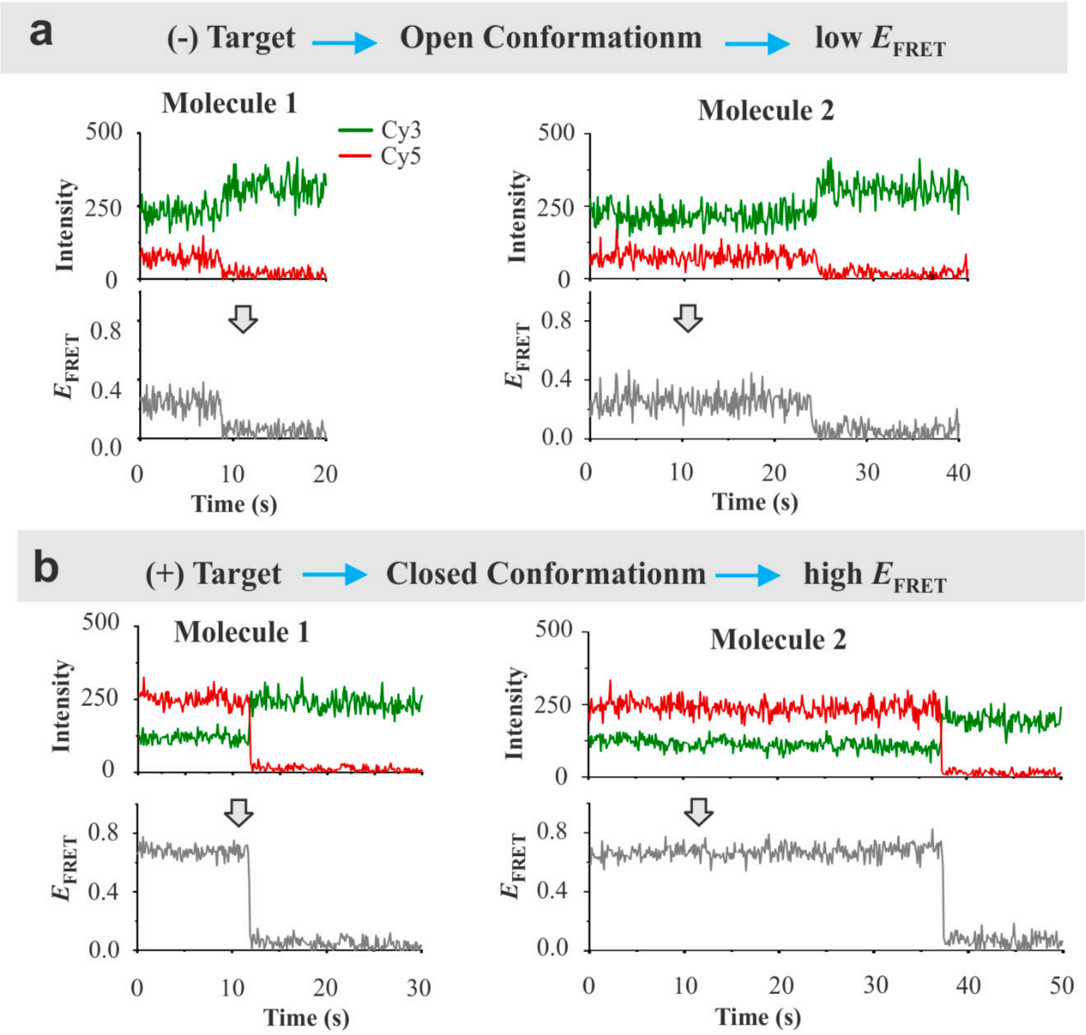

Figure S3. Typical single-molecule traces with photobleaching of fluorophores soon after illumination of green laser (<15 s, left panel) or after 20 s (right panel). Top panels display intensity-time traces of the Cy3 (green) and Cy5 (red) fluorophores and the bottom panels show corresponding FRET efficiencies. The single molecules were confirmed by anti-correlation between the Cy3 and Cy5 intensities. (**a**) Open conformation (low-E_{FRET} state). (**b**) Closed conformation (high-E_{FRET} state). All traces were taken from sensor-III.

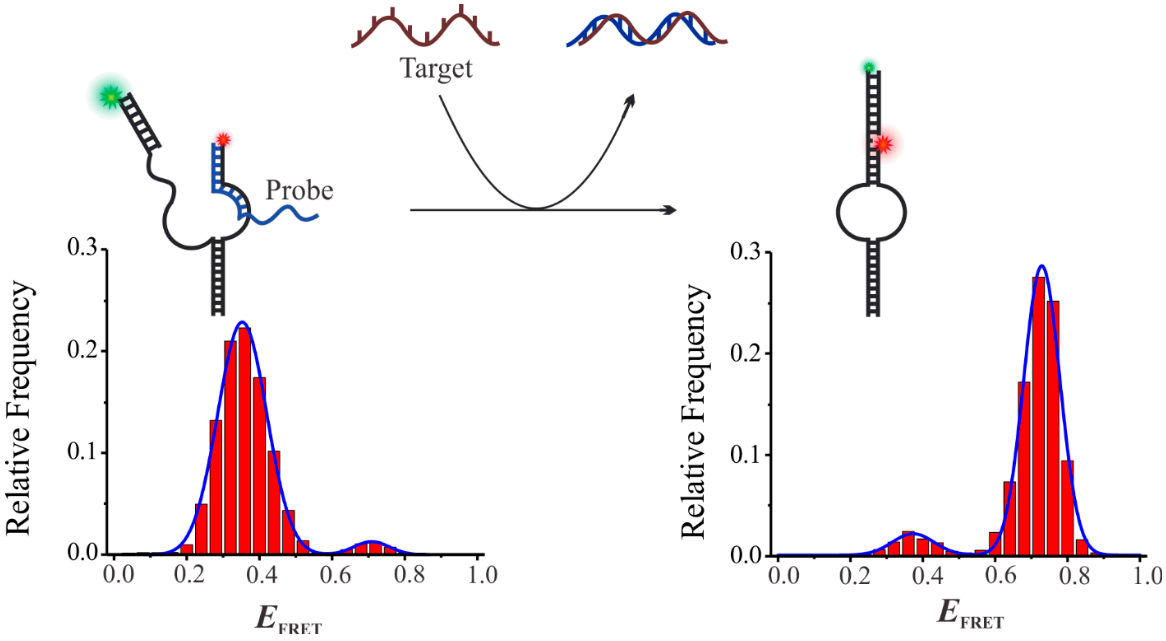

Figure S4. smFRET analysis of sensor-III with probe binding to Cy5-labeled strand. Unlike original design, the probe was designed to partially hybridized to the Cy5-labeled strand instead of Cy3-labeled strand. This experiment was performed to demonstrate the flexibility in the sensor design. As expected, in the presence of target, probe was displaced from the sensor; consequently, conformational switching of sensor-III resulted in high-E_{FRET} state. The experiment was performed at 1 μM target.

References

1. Kaur, A.; Sapkota, K.; Dhakal, S. Multiplexed Nucleic Acid Sensing with Single-Molecule FRET. *ACS Sens.* **2019**, *4*(3), 623–633.
2. Gibbs, D. R.; Dhakal, S. Single-Molecule Imaging Reveals Conformational Manipulation of Holliday Junction DNA by the Junction Processing Protein RuvA. *Biochemistry* **2018**, *57* (*26*), 3616–3624.
3. Newby Lambert, M.; Vöcker, E.; Blumberg, S.; Redemann, S.; Gajraj, A.; Meiners, J.-C.; Walter, N. G. Mg2+-Induced Compaction of Single RNA Molecules Monitored by Tethered Particle Microscopy. *Biophys J.* **2006**, *90* (*10*), 3672–3685.
